# Vitamin D Status: A Different Story in the Very Young versus the Very Old Romanian Patients

**DOI:** 10.1371/journal.pone.0128010

**Published:** 2015-05-29

**Authors:** Adela Chirita-Emandi, Demetra Socolov, Carmen Haivas, Anca Calapiș, Cristina Gheorghiu, Maria Puiu

**Affiliations:** 1 Genetics Department, University of Medicine and Pharmacy “Victor Babeș”, Timișoara, Romania; 2 Ginecology Department, University of Medicine and Pharmacy “Gr. T. Popa”, Iași, Romania; 3 Anatomy Department, University of Medicine and Pharmacy “Victor Babeș”, Timișoara, Romania; 4 Bioclinica Laboratoarele, SA, Timișoara, Romania; 5 Genetics Department, Clinical Emergency Hospital for Children “Louis Țurcanu”, Timișoara, Romania; Nanjing Medical University, CHINA

## Abstract

**Background:**

In Romania (latitude 48°15’N to 43°40’N), vitamin D supplementation is common practice mostly in infants 0-1 year old. No published information is available regarding epidemiological data on vitamin D status in the Romanian population for a wide age range and geographical territory. In this context, we aimed to evaluate the seasonal and age variation of vitamin D status in a large Romanian population.

**Methods:**

6631 individuals from across Romania had performed 7544 vitamin D assessments (2012-2014) in a chain of private laboratories. Vitamin D (25-hydroxyvitamin D2 and 25-hydroxyvitamin D3) was measured using High Performance Liquid Chromatography. Vitamin D levels were classified as severe deficiency<10ng/mL, deficiency 10-20ng/mL, insufficiency 21-29ng/mL, sufficiency≥30ng/mL and potentially harmful>100ng/ml.

**Results:**

Male to female ratio was 1:2.9. Age ranged from 0 to 85 years. Mean vitamin D levels increased from April (26.3ng/ml) to September (35.6ng/ml) and decreased from October (33.5ng/ml) to March (24.4 ng/ml). Overall 40% had sufficient vitamin D, while the rest were insufficient 33%, deficient 22%, severely deficient 4% and 1% potentially harmful (of them 81% under 1 year old). Males compared to females showed higher percentages of sufficiency (47% vs. 38%). Children 0- 2 years presented the highest percentage of vitamin D sufficiency (77%). Lowest percentages (21%) of sufficiency were in people 80-84 years.

**Conclusion:**

In Romania, suboptimal vitamin D levels are common (59%), especially in older age, wintertime and in women. Vitamin D supplementation would be most warranted from January to April in the Romanian population. 25-hydroxyvitamin D levels>100ng/ml were relatively prevalent in children 0-1 year old (17.3%). This was attributed to supplementation errors and the fact that high-risk individuals were more likely to visit for medical check-up. Nonetheless, it stresses the need to increase awareness of the importance of preventing Vitamin D supplementation administration errors in the young.

## Background

Vitamin D is a fat-soluble compound that plays a significant role in calcium homeostasis and bone metabolism. In recent years, aside from the role of vitamin D in rickets and osteomalacia, there has been a strong interest to link vitamin D insufficiency with increases in cardiovascular disease,[1,2] cancer,[3] asthma,[4] and infection.[5,6] In other studies, low vitamin D levels have also been linked with women’s reproductive health outcomes [7] and earlier age of menarche.[8] Vitamin D supplementation seems to alleviate the incidence or adverse outcomes of these diseases and may reduce all-cause mortality.[9]

The major circulating form of vitamin D is 25-hydroxyvitamin D, which is considered the best indicator of vitamin D status.[10] The prevalence of vitamin D deficiency varies greatly based on how deficiency is defined (<20 vs. ≤30 ng/mL). According to NHANES data, 8% of the population had very low 25-hydroxyvitamin D levels (<12 ng/mL), and 25% were at risk for deficiency (12 to 20 ng/mL).[11] A NHANES 2001 to 2004 study regarding prevalence of 25-hydroxyvitamin D levels of less than 30 ng/mL, showed that 77% of noninstitutionalized United States participants had 25-hydroxyvitamin D levels below 30 ng/mL.[12] In Europe, the HELENA study reported that about 80% of their sample had suboptimal levels (39% had insufficient, 27% deficient and 15% severely deficient levels). Vitamin D deficiency is highly prevalent in European adolescents and constitutes a public health issue.[13]

In Romania several studies evaluated the vitamin D status in some areas of the country, in certain groups such as elderly individuals in nursing homes,[14,15] in health caregivers,[16] in postmenopausal women,[17] in patients from an endocrinology clinic,[18] or in patients with inflammatory bowel disease.[19] However, no published information is available regarding epidemiological data on vitamin D status in a Romanian population for a wide age range and geographical territory. In this context, we aimed to evaluate the seasonal and age variation of vitamin D status in a large Romanian population.

## Material and Methods

### Subjects

This is a cross-sectional study, where a group of 6631 unique individuals had performed a total of 7544 vitamin D, 415 ionized calcium and 611 PTH assessments between 1^st^ January 2012 and 30^th^ August 2014 in a chain of private laboratories. The Bioclinica laboratories collected blood samples from 60 sites across the country in 10 major cities (Timisoara, Arad, Bistrita, Bucuresti, Constanta, Deva, Iasi, Targu Jiu, Oradea, Resita). All the blood samples were centralized in a single center in Timisoara. Date of assessment, date of birth, gender, the specialty of referring physician was available for each patient. We excluded 359 assessments for which there was no data on gender or date of birth. Additionally, another 5 cases were excluded because they were considered outliers for PTH level (above 500 pg/ml). The patients were from both urban and rural environments. The total population in Romania in the most recent 2011 census of populationwas 20 121 641 (51.4% females).[20] The patients were referred from hospital and private practices. Data on their diagnosis was not available. The people were white, living at latitudes ranging from 48°15’N at the northernmost point to 43°40’N in the southernmost point of Romania and with altitude ranging from 25 m -235 m in urban areas.

### Assessment method

Vitamin D (25-hydroxyvitamin D2 and 25-hydroxyvitamin D3) was measured using the High Performance Liquid Chromatography method (Chromsystems Instruments & Chemicals GmbH). Using the Endocrine Society Clinical Practice Guidelines, vitamin D deficiency was defined when serum vitamin D (25OHD) levels were < 20 ng/mL (50 nmol/l); insufficiency if the vitamin D levels were between 21 and 29 ng/ml (52.5–72.5 nmol/l), and sufficiency for vitamin D concentration ≥ 30 ng/ml (75 nmol/l), [10] while vitamin D levels <10 ng/mL (25 nmol/l) were considered severe vitamin D deficiency.[21] Levels between 100–150 ng/ml (250–375 nmol/l) were considered as possibly harmful, while levels above 150 ng/ml (375 mmol/l) were considered toxic.

Ionized Calcium was assessed using Ion-Selective Electrode method with a Medica EasyLyte analyser. Normal Ionized Calcium values were considered between 4.4–5.2 mg/dl (1,1–1.3 mmol/l).

Parathyroid hormone (PTH) was assessed by Electrochemiluminescence Immunoassay (Cobas, Roche Diagnostics). Serum PTH levels ≥ 65 pg/ml (7 pmol/l) were considered to be significantly elevated. Low PTH was considered when values were under 15 pg/ml (1.6 pmol/l).

“Winter” (insufficient sunlight) months were considered to be from October to March, while “summer” (abundant sunlight) months were from April to September.

### Statistical analysis

Descriptive statistics are outlined for all variables assessed. Means and SDs were calculated for all normally distributed continuous measures including age, vitamin D, ionized calcium and PTH level. Data were analyzed with SPSS 22 (SPSS Inc, Chicago). The independent samples T test was used for comparing variance. Pearson correlation was used for comparing means.

### Ethics

The study simply analyzed data that was routinely collected as part of the clinical care of the patients, for which patients or guardians signed an informed consent. Patient’s data were anonymized before statistical analysis. The study was approved by the Ethics Committee of the University of Medicine and Pharmacy “Victor Babes” Timisoara, Romania.

## Results

In our study, male to female ratio was 1:2.9. Mean age was 39.5 years decimal age (SD = 22.1), ranging from 0.03 to 84.9 years. Table 1 presents descriptive statistics of vitamin D, Ionized Calcium and PTH with number of cases, mean and standard deviation for all assessments, for each decade and for male and female separately. Values of p<0.05 in bold depict significant differences between male and female.

**Table 1:** Descriptive statistics with mean and standard deviation on vitamin D, ionized calcium and PTH in respect to decade of age and gender.

	Vitamin D ng/ml				Calcium mg/dl				PTH pg/ml			
Variables	N	Mean	SD	p	N	Mean	SD	p	N	Mean	SD	p
**All cases**	7544	27.20	16.76		415	4.90	0.36		611	66.73	51.12	
**All cases M**	1917	34.45	21.56	**0.000**	120	4.49	0.42	0.148	94	67.06	43.89	0.494
**All cases F**	5627	28.42	14.45		295	4.88	0.32		517	64.88	80.28	
**decade 1: age 0 to 10 years**	1199	45.31	26.89		74	5.15	0.32		28	65.52	107.98	
**decade 1 M**	694	45.73	27.78	0.523	43	5.09	0.36	**0.080**	19	78.78	129.40	**0.024**
**decade 1 F**	505	44.74	25.64		31	5.22	0.26		9	37.52	19.40	
**decade 2: age 11 to 20 years**	564	25.52	10.47		58	4.89	0.42		24	28.95	18.52	
**decade 2 M**	285	25.39	9.71	0.770	26	5.00	0.22	**0.054**	11	33.75	13.99	0.108
**decade 2 F**	279	25.65	11.20		32	4.80	0.52		13	24.90	21.34	
**decade 3: age 21 to 30 years**	573	26.13	10.78		41	4.86	0.14		39	56.64	42.77	
**decade 3M**	121	25.81	10.47	0.711	10	4.91	0.17		14	73.94	58.28	0.067
**decade 3 F**	452	26.21	10.87		31	4.85	0.13	0.276	25	46.96	28.00	
**decade 4: age 31 to 40 years**	1077	26.77	12.33		58	4.84	0.22		68	53.86	25.13	
**decade 4 M**	209	28.92	13.59	**0.010**	12	4.88	0.21	0.501	7	37.90	9.98	0.231
**decade 4 F**	868	26.26	11.95		46	4.83	0.22		61	55.69	25.73	
**decade 5: age 41 to 50 years**	1189	27.58	12.80		70	4.87	0.25		94	61.33	45.91	
**decade 5 M**	226	32.20	17.93	**0.000**	13	4.87	0.18	0.861	10	80.59	118.04	**0.000**
**decade 5 F**	963	26.50	10.99		57	4.86	0.27		84	59.04	28.30	
**decade 6: age 51 to 60 years**	1388	27.85	11.73		63	4.82	0.47		168	71.41	50.81	
**decade 6 M**	171	29.02	12.70	0.196	10	4.41	1.04	0.174	16	45.09	26.86	0.219
**decade 6 F**	1217	27.69	11.58		53	4.90	0.20		152	74.18	51.99	
**decade 7: age 61 to 70 years**	1030	27.83	12.00		35	4.77	0.43		134	78.22	52.33	
**decade 7 M**	130	27.79	12.23	0.966	5	4.84	0.13	0.467	12	65.14	72.23	0.850
**decade 7 F**	900	27.84	11.98		30	4.76	0.46		122	79.51	50.17	
**decade 8: age 71 to 80 years**	480	25.72	12.60		12	4.99	0.43		52	73.66	41.84	
**decade 8M**	73	26.06	13.25	0.806	1	5.52		NA	5	124.19	51.96	0.190
**decade 8 F**	407	25.65	12.49		11	4.94	0.42		47	68.29	37.41	
**decade 9: age 81 to 90 years**	44	22.16	15.26		4	4.86	0.20		4	74.39	29.07	
**decade 9 M**	8	18.55	10.25	0.343	0			NA	0			NA
**decade 9 F**	36	22.96	16.17		4	4.86	0.20		4	74.39	29.07	

N = number of assessments; SD = standard deviation; p value from Student T test.

Children (0 to 18 years) were referred for vitamin D assessment by paediatric endocrinologists in 48% of cases, general paediatricians in 24%, orthopaedics, rheumatology and dental medicine in 3.5% and from other specialists in 24.5% of cases. In adults, most (51%) cases were referred by the endocrinologists, 10.5% by the general practitioner, 5% by internal medicine specialists, 5% by nephrologists, 3.5% by rheumatologists and 25% by other specialists.

### Vitamin D status seasonal variation

**Fig 1:**
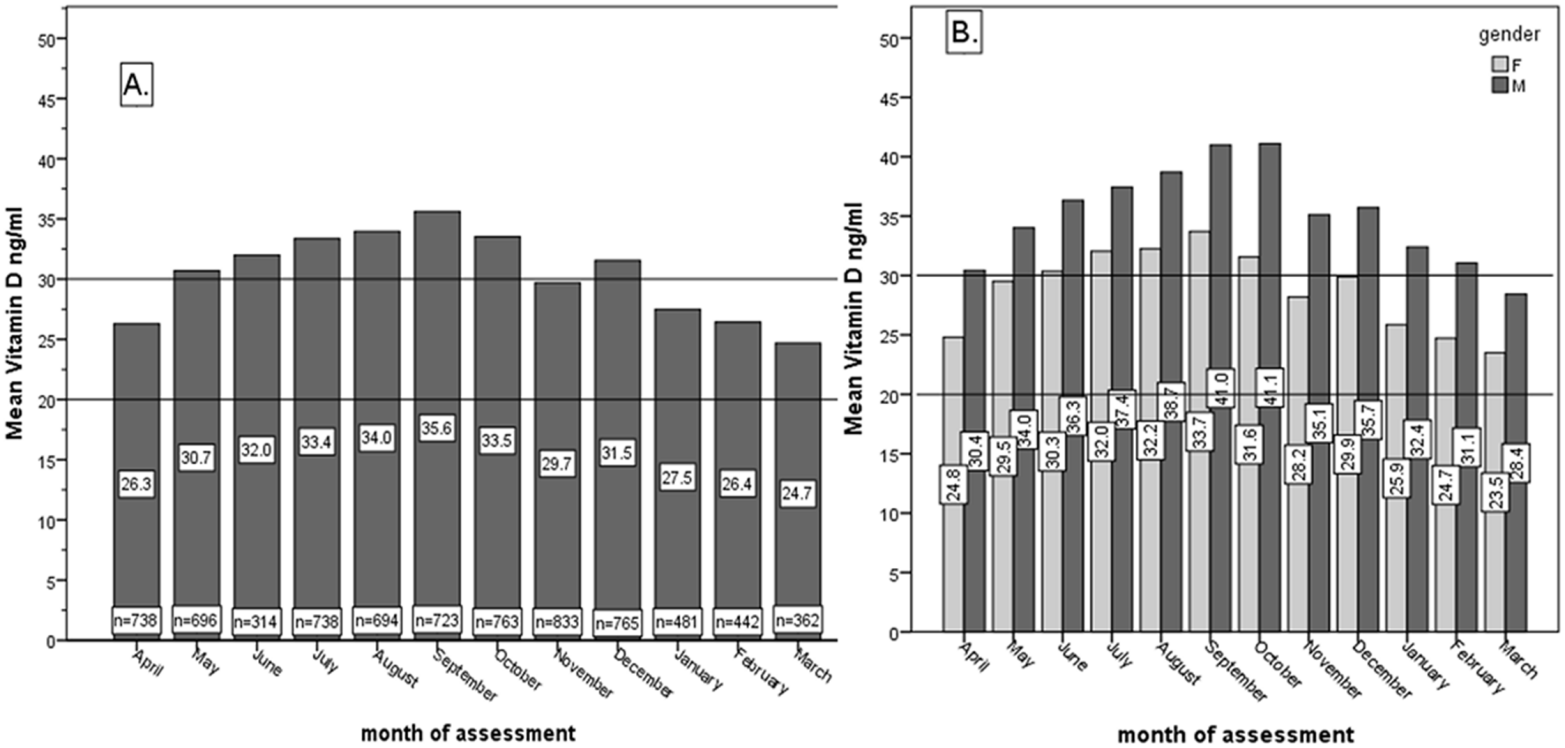
A. Overall mean vitamin D (ng/ml) with regards to month of assessment. B. Female and male comparison of mean vitamin D value (ng/ml) with regards to month of assessment.

Fig 1 presents the mean vitamin D (ng/ml) of all assessments with regards to month of assessment (A) and separately the female and male comparison of mean vitamin D value with regards to month of assessment (B). There was a slow increase in mean vitamin D levels from April (26.3ng/ml) to September (35.6ng/ml) and a decreasing trend from October (33.5ng/ml) to March (24.4 ng/ml). Males showed larger mean vitamin D values compared to females on age decades. Male to female difference in mean was in average 6.3 ng/ml, being greatest in October (9.5ng/ml). The difference was statistically significant (p value ranging from 0.000 to 0.047) for all months. Considering there were more females than males, we tried to equalize the groups, by randomizing the women into three groups of 1919 vitamin D assessments. The mean values difference between the randomized groups compared to the entire female group was less than 0.8 ng/ml in each case, the difference between male and female with regards to mean vitamin D was maintained throughout the randomized groups’ analysis.

**Fig 2:**
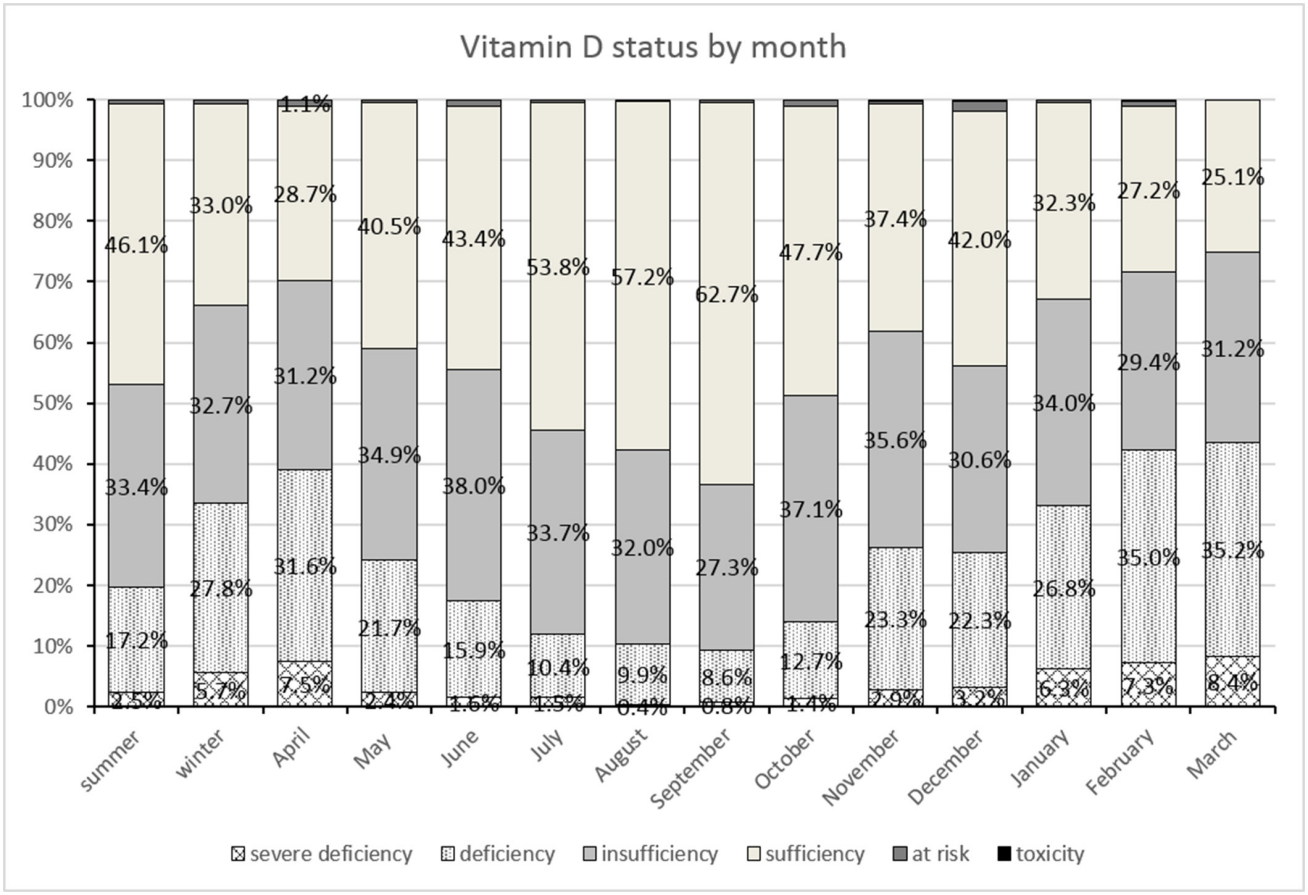
Percentages of vitamin D status, classified as severe deficiency, deficiency, insufficiency, sufficiency, “possibly harmful” and toxicity for all assessments, for summer and winter seasons and separately for each assessment month.

Fig 2 illustrates the percentages of vitamin D status, classified as severe deficiency (vitamin D <10 ng/mL), deficiency (between 10 and 20 ng/mL), insufficiency (between 20 and 30 ng/mL), “possibly harmful” (between 100 and 150 ng/mL) and toxicity (>150 ng/mL).[10] One column for summer, one for winter and separately for each month is presented. High percentages of vitamin D sufficiency were seen from June (43.4%) to October (47.7%), being highest in September (62.7%). The lowest percentage (25%) for sufficient vitamin D status was seen in March. Conversely, the highest percentages for deficiency and severe deficiency were observed also in March at 35.2% and 8.4% respectively. Toxicity and “possibly harmful” percentages did not have a particular distribution with regards to month of vitamin D assessment.

### Vitamin D status variation with age

**Fig 3:**
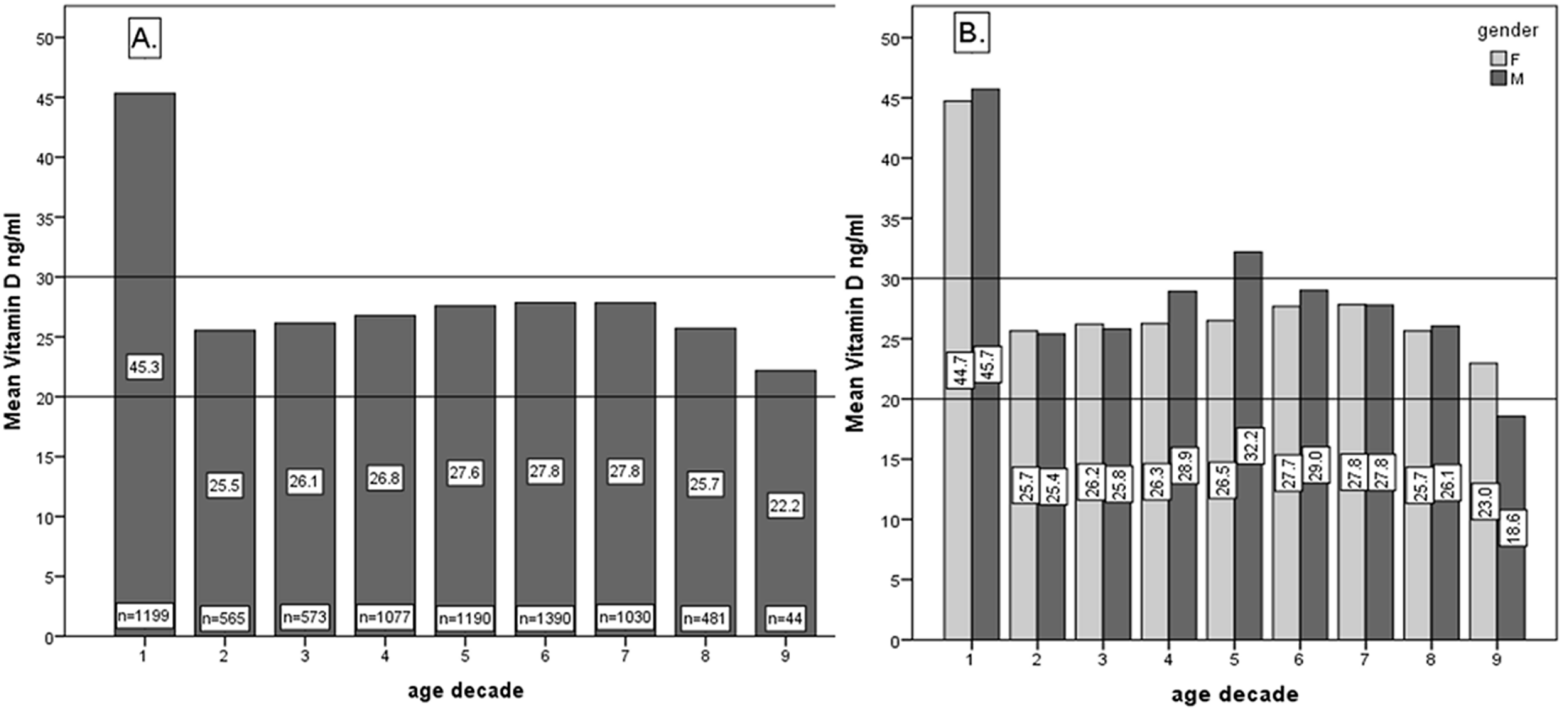
A. Overall mean vitamin D (ng/ml) with regards to age decade. B. Female and male comparison of mean vitamin D value (ng/ml) with regards to age decade. Age decade 1 includes ages 0 to 10 years, age decade 2 includes ages 11 to 20 years and so forth.

Fig 3 shows the mean vitamin D (ng/ml) with regards to age decade and separately, the female and male comparison of mean vitamin D with regards to age decade. There was a significant difference between the first age decade (mean 45.3 ng/ml) and subsequent decades (mean ranging from 27.8 to 22.2 ng/ml). In detailed age analysis within a decade, major dissimilarities between ages were noticed only in the first decade. Considerably higher values of mean vitamin D were seen in the 0 to 3 years group (data not presented in fig): age 0 to 1 years mean 68.5 ng/ml (SD = 38.2); age 1 to 2 years mean = 64.9 ng/ml (SD = 29.9); age 2–3 years = 38.2 ng/ml (SD = 20.9); versus a mean below 33.8 ng/ml for ages older than 3 years.

**Fig 4:**
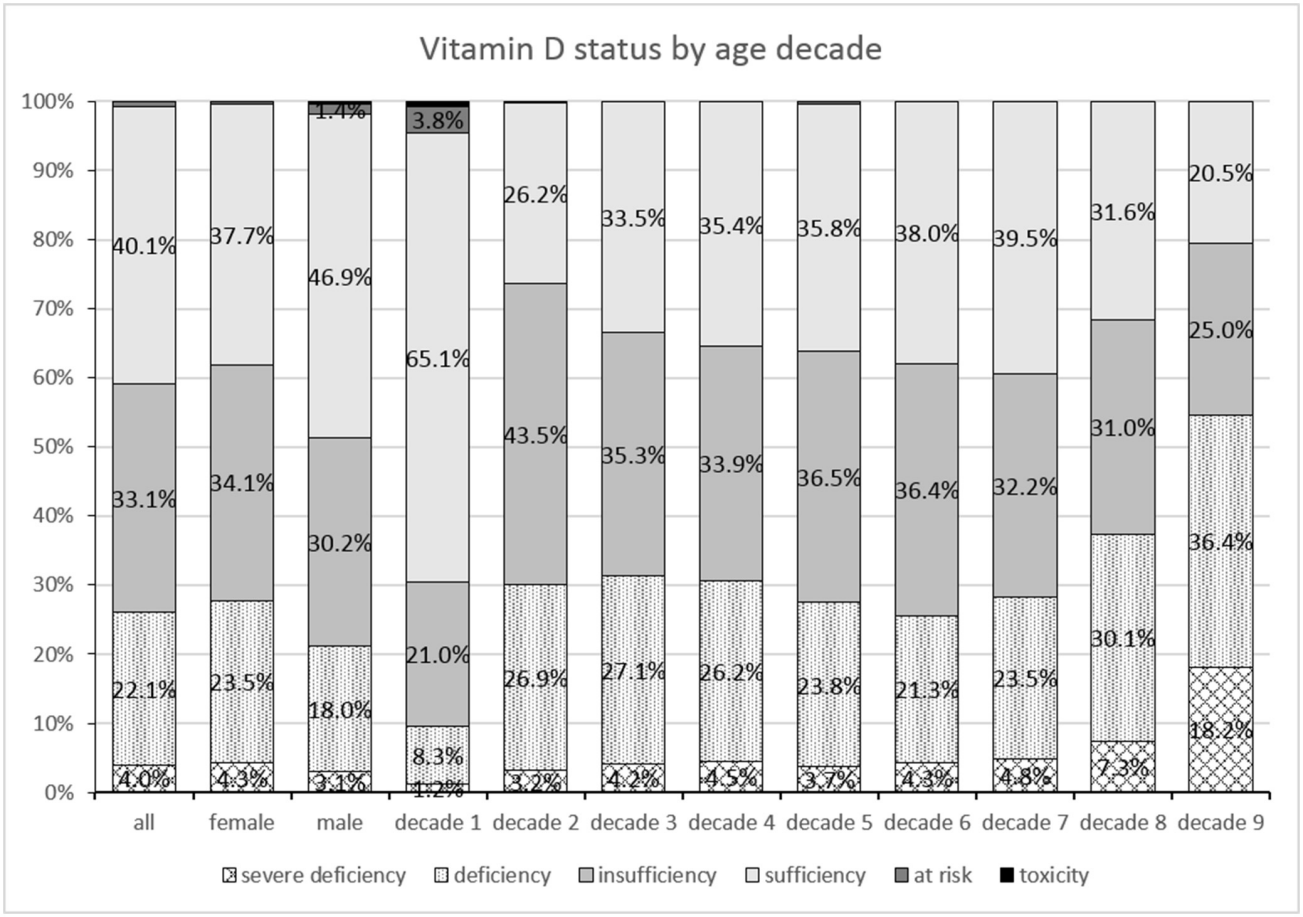
Percentages of vitamin D status, classified as severe deficiency, deficiency, insufficiency, sufficiency, “possibly harmful” and toxicity for all assessments, for female and male and separately for each age decade. Age decade 1 includes ages 0 to 10 years, age decade 2 includes ages 11 to 20 years and so forth.

The percentage distribution of vitamin D status is presented in Fig 4. with regards to gender and age decade. Overall 40.1% had a sufficient level of vitamin D, 33.1% presented insufficiency, 26.1% were deficient and 0.8% had levels above 100 ng/ml. Overall ages, when compared to females, males show higher percentages of sufficiency (46.9% versus 37.7%) but also higher rates for “possibly harmful” (1.4% versus 0.4%) and for toxicity (0.4% versus 0%).

Decade 1 differs considerably in comparison to the other decades, having the highest percentage (65.1%) for sufficient vitamin D, but also having vitamin D levels above 150ng/ml in 0.7% of assessments. In the 0 to 3 years group (data not presented in fig): age 0 to 1 year (n = 75) levels were: sufficient 68%, insufficient 5.3%, deficient 5.3%, severely deficient 4%, possibly harmful 13.3% and toxic 4%. In age 1 to 2 years (n = 320) levels were: sufficient 84.1%, insufficient 3.8%, deficient 1.6%, severely deficient 0%, possibly harmful 9.1% and toxic 1.6%. In age 2–3 years (n = 196) levels were: sufficient 33.5%, insufficient 19.9%, deficient 1.5%, severely deficient 0.5%, possibly harmful 2% and toxic 0%.

There seems to be a decreasing trend of vitamin D sufficiency, alongside growing rates of insufficiency and deficiency with increasing age decade. In the age group 80 to 85 years (decade 9), there were only 44 assessments, however this group presented the highest rates for deficiency and severe deficiency, 36.4% and 18.2% respectively.

### Vitamin D in relation to Ionized Calcium and PTH

Vitamin D had a poor positive Pearson correlation with ionized calcium (R = 0.180; p = 0.000), and weak negative correlation with the PTH (R = -0.164; p = 0.000). The ionized calcium correlated with PTH values (R = 0.493; p = 0.000).

Regarding calcium status, 53 assessments (12.8%; 30 in females) presented hypercalcemia, while 16 assessments (3.9%; 12 in females) presented hypocalcemia. High PTH values were noted in 233 assessments (37.8%; 212 in females), while 22 assessments (3.6%; 16 in females) presented low PTH values.

Out of those assessments that were deficient or severely deficient in Vitamin D, 94 also had calcium levels assessed, n = 10 (10.3%) presented hypercalcemia, 3 (3.1%) had hypocalcemia and 84 (86.6%) had normal ionized calcium values.

**Fig 5:**
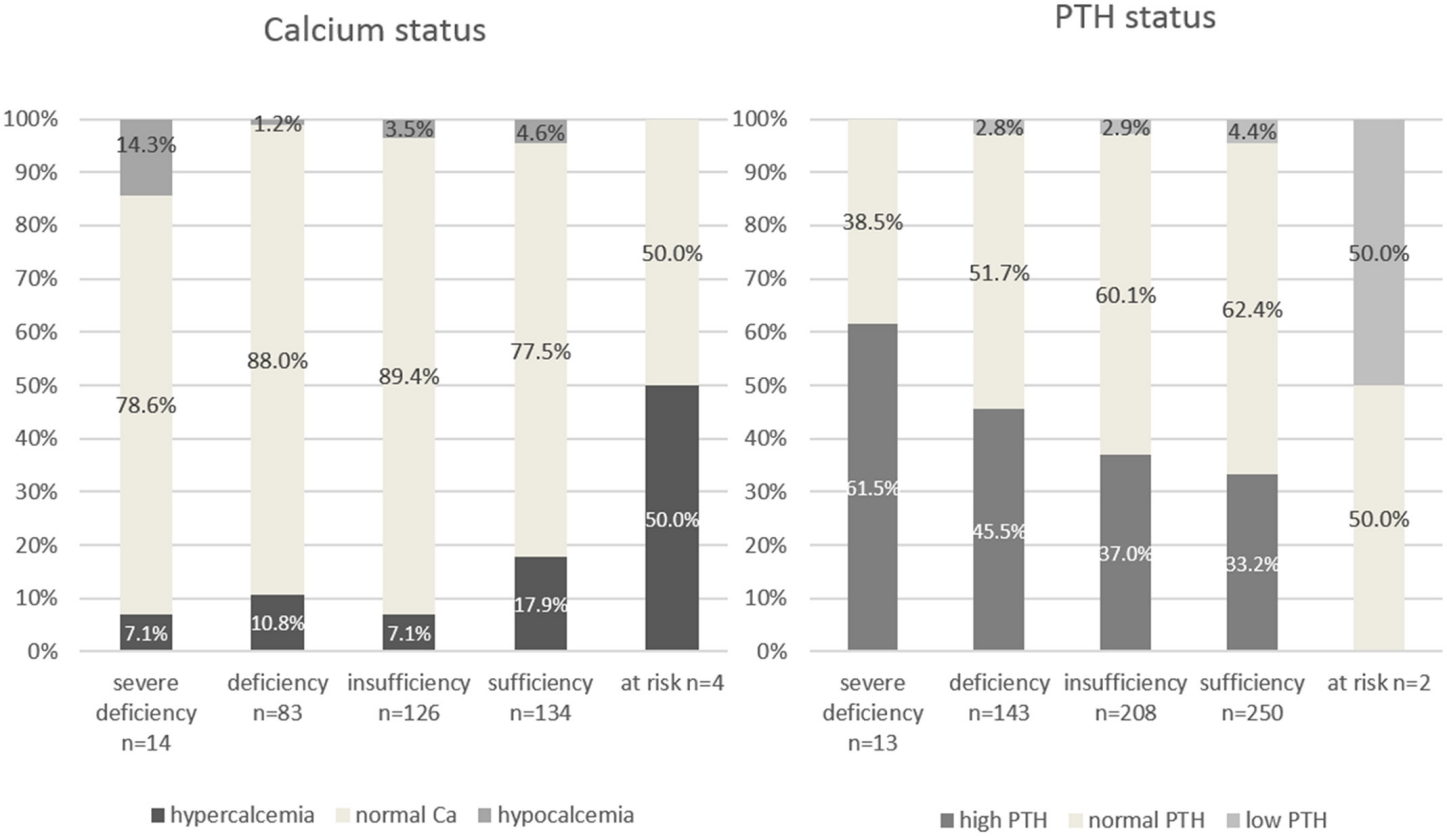
Percentages for ionized calcium and PTH status in regards to vitamin D status. Number of assessments for Calcium and PTH status are provided underneath each column.

Percentages for ionized calcium and PTH status with regards to vitamin D status are presented in Fig 5. Hypocalcemia was present mostly in the severe vitamin D deficiency class, while hypercalcemia was present in all vitamin D classes, with higher values in the sufficiency group. High PTH was seen in severe deficiency, deficiency and vitamin D insufficiency with rates ranging from 51.5% to 37%, but also in the sufficiency group in 33.2% of assessments.

The relation between Calcium and PTH levels was not evaluated because both were available for only 61 cases.

## Discussion

### Vitamin D status cut-offs

Presently, there is no international consensus on optimal 25-hydroxy vitamin D concentrations. To determine sufficiency cut-off levels, researchers have examined the level of 25-hydroxy vitamin D in relation to maximal suppression of parathyroid hormone, maximum calcium absorption, and reduced fracture risk. Experts generally agree that levels lower than 20 ng/mL (50 nmol/L) may be possibly harmful for bone health.[22] In 2011, the Institute of Medicine (IOM) concluded that 20 ng/mL was the level necessary for good bone health for practically all individuals.[23] In contrast, the Endocrine Society, National Osteoporosis Foundation, and International Osteoporosis Foundation suggest that 25-hydroxy vitamin D levels should be greater than 30 ng/mL (75 nmol/L), particularly in older adults. The Endocrine Society proposes targeting a higher level as it would better ensures that all persons meet target levels, considering the variability in laboratory measurements of 25-hydroxy vitamin D. The IOM concluded, however, that there may be a potential U-shaped relationship between 25-hydroxy vitamin D and some outcomes that pose potential health risks (e.g., mortality, cardiovascular disease, selected cancers, falls) at levels higher than 50 ng/mL (125 nmol/L).[23] Experts agree that optimal serum 25-hydroxy vitamin D concentrations for extra-skeletal health have not been established.[10,22,23] In this study we used the cut-offs proposed by the Endocrine Society.

### Vitamin D status seasonal variation

In the Romanian population, we found an increasing trend in mean vitamin D levels from April (26.3ng/ml) to September (35.6ng/ml) and a decreasing trend from October (33.5ng/ml) to March (24.4 ng/ml), Fig 1. Similarly, we found high percentages of vitamin D sufficiency in summer months, being highest in September (62.7%). The lowest percentage (25%) for sufficient vitamin D status was seen in March. The highest percentages for deficiency and severe deficiency were seen also in March, being 35.2% and 8.4%, respectively. Considering Romania’s latitude (48°15’N to 43°40’N) and seasonal sun exposure, March is the month that ends the winter season (insufficient light), thus this Vitamin D pattern is to be expected. A longitudinal study in Central Europe reported wintertime 25-hydroxy vitamin D values close to 21–23 ng/mL for all studied age groups, with a significant increase of 25-hydroxy vitamin D in August reaching 42 ng/mL for those aged 0–9 years, but only 21 ng/mL for the elderly aged 80–89 years.[24] Thus, vitamin D supplementation would be warranted mostly from January to April in the Romanian population.

As “possibly harmful” levels of vitamin D are not in relation to sun exposure, but to vitamin D supplementation issues, we did not observe a particular seasonal distribution of “possibly harmful” levels of vitamin D.

Our study identified mean vitamin D levels generally higher among males as compared to female. Male to female difference in mean was on average 6.3 ng/ml, possibly linked to increased use of sunscreen in women, less time spent outdoors and clothing particularly among females. Other studies have also found that females had greater risk for vitamin D deficiency. [25–27]. In contrast, Absoud et al. did not find a significant gender difference in mean vitamin D levels, in children. [28]

### Vitamin D status variation with age

Considering the cut-offs from the Endocrine Society, this study determined an overall percentage of 40% for sufficient level of vitamin D, 33% for insufficient, 26% for deficient and 1% for possibly harmful and toxic level, in the Romanian population. A Romanian study on 440 patients in an endocrinology clinic, showed only 12% with sufficient levels of vitamin D, possibly because high-risk individuals were more likely to have been to admitted in the clinics.[18] The HELENA study in adolescents reported 80% of their sample with suboptimal levels (39% insufficient, 27% deficient and 15% severely deficient)[13],while in our study we found suboptimal levels in 66% (35% insufficient, 27% deficient, 4% severely deficient) in the third age decade group. The percentages are similar, except for severe deficiency.

Several studies demonstrated that mean levels of 25-hidroxy vitamin D decrease with age for both males and females and that the prevalence of deficiency generally increased with age.[24,29,30] In this study, there was no clear descending trend of mean vitamin D levels with age decade (except for decade 8 and 9 where mean vitamin D was significantly decreased). Gill et al have also found that age was not significantly associated with mean vitamin D levels.[31] However, we did find a decreasing trend of percentages of vitamin D sufficiency, alongside growing rates of insufficiency and deficiency with increasing age decade. Vitamin D sufficiency was sporadic in older age (31% for age 70 to 80 years, 25% for age 80 to 84 years). The decrease in 25-hydroxy vitamin D with respect to age was attributed to decreased time spent in the sun and decreased vitamin D production efficiency. Aging decreases the capacity of human skin to produce vitamin D3.[30] Based on the literature review on vitamin D status in the Central Europe populations, it can be concluded that 25-hydroxy vitamin D levels are on average below the 30 ng/mL level.[24] Vitamin D3 and calcium supplementation may decrease the incidence of hip and other peripheral fractures. Vitamin D3 is recommended in housebound elderly, and it may be cost-effective in hip fracture prevention in selected risk groups.[32,33]

This study identified significantly greater mean vitamin D levels in the first age decade (45.3 ng/ml) compared to the subsequent decades (27.8 to 22.2 ng/ml). Children in the first decade had the highest percentage (65%) for sufficient vitamin D, but also had vitamin D levels above 150 ng/ml in 0.7% of assessments. In detailed analysis we found considerably higher values of mean vitamin D in the 0 to 2 years group (age 0 to 1 year mean 68.5 ng/ml; age 1 to 2 years mean = 64.9 ng/ml) compared to older ages. This was attributed to supplementation errors and the fact that high-risk individuals were more likely to visit for medical check-up. Nonetheless, it stresses the need to increase awareness of the importance of preventing Vitamin D supplementation administration errors in young age.

The safe upper value for 25-hydroxy vitamin D for avoiding hypercalcemia is not known, however, most studies in children and adults have suggested that the blood levels below 150 ng/ml should not be harmful. Therefore, an upper limit (UL) of 100 ng/ml provides a safety margin in reducing risk of hypercalcemia.[34] The high rates for “possibly harmful” or toxic vitamin D levels in the first two years of life, in our study, could be attributed to errors in administering the vitamin D supplements (D2 or D3) or to intra- and interindividual variation in drug-metabolising enzyme activity. During growth and development, changes in drug-metabolising enzyme activity result in age-related differences in drug disposition.[35] Currently, in Romania, a consensus for vitamin D supplementation recommendations for infants does not exist. Recommendations regarding supplementation usually vary between 400–800 IU/day in the first year of life. Some paediatricians recommending supplementation for the first two years and then afterwards in winter months. To our knowledge, there is no study in Romania looking at maternal practices and awareness of vitamin D supplementation in infants. The IOM report [23] recommended that “the tolerable UL for vitamin D should be 1000 IU /d for children 0–6 months, 1500 IU /d for children 6 months to 1 years, 2500 IU /d for children 1–3 years, and 3000 IU /d for children 4–8 years”.[10] For children older than 9 years and adults, IOM recommended UL of 4000 IU /d. [10]

### Vitamin D in relation to Ionized Calcium and PTH

Vitamin D and PTH both work to provide calcium homeostasis in the blood. PTH is secreted when calcium levels decrease. PTH stimulates release of calcium from bone into blood and synthesis of 1,25 dihydroxy vitamin D from its storage form 25hydroxy vitamin D.[36,37]

In our study, vitamin D had a poor positive Pearson correlation with ionized calcium (R = 0.180,p = 0.000), and weak negative correlation with the PTH (R = -0.206, p = 0.000). The ionized calcium correlated with PTH values (R = 0.493). Similar correlation values have been found by Vierucci et al.[38]

We found that 13% of ionized calcium assessments presented as hypercalcemia, while 4% presented as hypocalcemia. High PTH values were noted in 37% of assessments. Azab et al found similar percentages for secondary hyperparathyroidism (33%).[39] Hypocalcemia was mostly associated with severe vitamin D deficiency, while hypercalcemia was more prevalent in people with sufficient and possibly harmful levels of Vitamin D. High PTH was seen in severe deficiency, deficiency and vitamin D insufficiency, but also in the sufficiency group in 36% of assessments.

Nonetheless, regarding the recommendation to perform vitamin D assessments, Smith et al concluded in their study that “routine measurements of calcium, phosphate, and alkaline phosphatase were not reliable predictors of hypovitaminosis D, even when vitamin D insufficiency has been sufficient to produce a PTH response”. [40] Authors recommend that the gold standard for assessing vitamin D should be clinical suspicion, medical history and an awareness of risk factors.[40]

### Limitations

There were significantly more women than men (3:1) in the study. This could be attributed to the fact that more women seek medical attention and perform more assessments compared to male.[41] An additional limitation is that we had no information on the pregnancy status of the women, thus, we could not draw any conclusion related to vitamin D in pregnancy. None of the patients with vitamin D toxicity had assessments for ionized calcium or PTH at these laboratories. At the time of the study, vitamin D and PTH assessments were not available in many public hospitals, thus, these assessments were performed in private laboratories. Conversely, ionized calcium assay was available in all hospital laboratories, thus very few assessments were made at Bioclinica. This is a cross-sectional study where patients were referred by their treating physicians for assessment, hence, it cannot be excluded that high-risk individuals were more likely to visit for medical check-up. Associated factors influencing vitamin D (including medication) were not assessed in this study.

## Conclusions

Our results suggest that suboptimal vitamin D levels are common in the Romanian patient population. Risk factors for vitamin D deficiency identified in this study were:older age, female gender and winter season. Vitamin D supplementation would be most warranted from January to April in the Romanian patient population. Further investigation is warranted to evaluate factors associated with vitamin D deficiency in high-risk individuals.

25-hydroxyvitaminD levels>100ng/ml were relatively prevalent in children 0–1 year old(17.3%). This was attributed to supplementation errors and the fact that high-risk individuals were more likely to visit for medical check-up. Nonetheless, it stresses the need to increase awareness of the importance of preventing Vitamin D supplementation administration errors in young age.

## Supporting Information

S1 File Raw individual data of vitamin D, PTH and Calcium levels for each individual. Identification number, date of assessment, season of assessment, gender, date of birth (month and year), decimal age are also provided.
(XLSX)
